# Evaluating the Effectiveness of Shockwave Lithotripsy vs Ureteroscopic Lithotripsy for Treatment of Renal Stones: A Prospective Cohort Study

**DOI:** 10.7759/cureus.75876

**Published:** 2024-12-17

**Authors:** Naeem Ahmed Butt, Syeda Wajiha Batool, Aiza Ali Akbar, Muhammad Amir, Yasir Qayyum, Amna Akbar, Marriam Khan, Hasnain Ali

**Affiliations:** 1 Department of Urology, Abbas Institute of Medical Sciences, Islamabad, PAK; 2 Medicine, Azad Jammu Kashmir Medical College, Muzaffarabad, PAK; 3 Medicine, Midland Doctors Medical Institute, Muzaffarabad, PAK; 4 Department of Surgery, Sheikh Khalifa Bin Zaid Hospital (SKBZ)Combined Military Hospital (CMH) Rawalakot, Rawalakot, PAK; 5 Emergency and Accident, District Headquarter (DHQ) Hospital, Jhelum, Muzaffarabad, PAK; 6 Health, Indus Hospital, Karachi, PAK; 7 Medicine, Army Medical College, Rawalpindi, PAK

**Keywords:** complications, prospective cohort study, recurrence, renal stones, shockwave lithotripsy, stone clearance, ureteroscopic lithotripsy

## Abstract

Background: Renal stones are a prevalent condition requiring effective treatment strategies. This study evaluates the comparative effectiveness of shockwave lithotripsy (SWL) and ureteroscopic lithotripsy (URSL) in treating renal stones in a Pakistani cohort, focusing on stone clearance, recurrence, and complications.

Methods: This prospective cohort study involved 250 patients aged 18-55 diagnosed with renal stones. Participants were divided into two groups based on treatment selection (SWL or URSL) and followed for 12 months. Primary outcomes included stone clearance rates, while secondary outcomes encompassed recurrence and complications. Statistical analyses, including chi-square tests and Mann-Whitney U tests, assessed demographic and clinical characteristics, with significance set at p < 0.05.

Results: The mean age of participants was 35.05 years, with an equal distribution between SWL and URSL groups. Both treatments demonstrated similar stone clearance rates, with no significant difference in success (p > 0.05). Complications were noted in 64.4% of cases, with bleeding (34.0%) and infection (30.4%) as the most frequent issues. Recurrence rates were comparable (p = 0.800), affecting 49.2% of patients. Re-treatment was required in 51.2% of cases, equally distributed between the groups.

Conclusion: SWL and URSL exhibit comparable effectiveness in treating renal stones, with similar success and recurrence rates. While complications were common, they were manageable. Both approaches are effective options for renal stone management, emphasizing the need for individualized treatment decisions based on patient and stone characteristics.

## Introduction

Kidney stones, also known as renal stones, are a significant public health issue worldwide. The formation of these stones is influenced by genetic, nutritional, atmospheric, and metabolic factors. They can cause severe pain, infections, and loss of kidney function if left untreated. The management of renal stones involves relieving symptoms, removing the stones, and preventing their recurrence. Two common procedures used in managing renal stones are shockwave lithotripsy (SWL) and ureteroscopic lithotripsy (URSL), which are less invasive and have their advantages and disadvantages [1, 2]. The supersaturation of substances in urine, particularly calcium, oxalate, and uric acid, causes the formation of renal stones. Factors contributing to the formation of renal stones include dehydration, an unbalanced diet, metabolic disorders, hereditary factors, and certain drugs. The population of patients with renal stones is growing rapidly in developed countries where obesity and metabolic syndrome are underlying causes [3]. Kidney stones tend to recur, with studies showing that about 50% of those with stones will have a recurrence within five years. This high recurrence rate calls for practical and non-invasive solutions to their management. Despite both SWL and URSL being acknowledged methods of renal stone management, there is still controversy over which method is more effective and safer. SWL uses special sound waves to fragment stones, but it has limitations when dealing with larger or denser stones [4]. UR requires percutaneous insertion of a ureteroscope through the urethra and bladder, using other tools like lasers to view and cut up the stones. However, URSL generally has better chances of successfully removing any stone from the patient's body, particularly when a stone is lodged in the lower region of the kidney or in the ureter [5].

Literature on the subject has highlighted variables such as the dimensions, location, and structure of the stones, as well as the age, anatomy, and history of a particular patient. Some studies suggest that URSL may achieve a higher stone-free rate, particularly in difficult cases, but SWL is still largely preferred due to its non-invasion and short duration of the procedure [6]. This research aims to evaluate and compare the effectiveness of SWL and URSL for managing renal stones, focusing on stone-free rates, complications, and recovery metrics. The study will also analyze factors such as infection, bleeding, and the need for reoperation after the procedures [7-9]. Additionally, patient-reported outcomes and quality of life will be measured to help patients make an informed decision regarding which treatment technique offers effective renal stone management.

## Materials and methods

Research design

This prospective cohort study aimed to compare the effectiveness of SWL and URSL in treating renal stones in Pakistani patients aged 18 to 55 years. The study followed patients for a period of 12 months to evaluate the primary outcome of stone clearance, along with secondary outcomes such as recurrence rates and complications. Patients were allocated into two treatment groups based on their choice of intervention (SWL or URSL) and were monitored over the follow-up period to assess the efficacy and safety of the two procedures.

Inclusion and exclusion criteria

The inclusion criteria for the study were as follows: patients aged 18 to 55 years who had been diagnosed with renal stones through CT or ultrasound and who had opted for either SWL or URSL as their treatment method. Exclusion criteria included individuals with recurrent stone disease requiring surgical intervention, pre-existing urological abnormalities (such as congenital malformations or strictures), or contraindications to either SWL or URSL (e.g., pregnancy, active urinary tract infections, or severe renal dysfunction). These criteria ensured that the sample was homogenous and that the results would be generalizable to the targeted population.

Sampling technique

A consecutive sampling technique was utilized to recruit participants for the study. Eligible patients who presented to the urology department of a tertiary care hospital and met the inclusion criteria were enrolled in the study after providing informed consent. This approach ensured that the study sample was representative of the general population seeking treatment for renal stones.

Study location and duration

The study was conducted at a tertiary care hospital in Lahore, Pakistan, over the period from January 2023 to December 2023. During this time, patients underwent either SWL or URSL as part of their standard care, and follow-up was conducted for 12 months post-treatment. The follow-up period allowed for comprehensive monitoring of the primary and secondary outcomes, including stone clearance, recurrence, and any complications associated with the treatments.

Statistical analysis

Data analysis was performed using SPSS version 26. Descriptive statistics were used to summarize the baseline demographic and clinical characteristics of the study population, providing an overview of key variables such as age, gender, and stone size. The chi-square test was employed to compare categorical variables between the SWL and URSL groups, while the Mann-Whitney U test was used for continuous variables to assess differences in stone size and other clinical parameters. To examine the relationship between treatment and outcomes (stone clearance, recurrence, and complications), logistic regression models were applied, adjusting for potential confounders such as age, stone size, and comorbidities. A significance level of p<0.05 was used to determine the statistical significance of the results.

Ethical considerations

The study was approved by the Institutional Review Board (IRB) of Abbas Institute of Medical Sciences Muzaffarabad. Written informed consent was obtained from all participants before their inclusion in the study. The participants were fully informed about the nature of the study, its procedures, and the potential risks and benefits. The study adhered to the ethical principles outlined in the Declaration of Helsinki, ensuring the protection of participants' rights, confidentiality, and privacy throughout the research process.

## Results

This prospective cohort study was designed to assess the relative effectiveness of SWL compared to URSL in treating renal stones among a Pakistani population aged 18 to 55. This section outlines the demographic and clinical features of the study participants, along with a comparison of the treatment outcomes associated with the two lithotripsy techniques.

Demographic and clinical characteristics

The study comprised 250 participants, with a slight predominance of males (52.4%) over females (47.6%). The mean age of the participants was 35.05 years (SD = 10.55). Body Mass Index (BMI) varied from 18.7 to 34.9, averaging 26.93, which indicates a diverse range of normal and elevated BMI levels within the group. Furthermore, 52.8% of the participants were identified as smokers, and 51.6% reported consuming alcohol, suggesting lifestyle factors that may contribute to the development or recurrence of renal stones. This information is detailed in Table 1. The presence of comorbidities was notable, with around 75.2% of participants indicating at least one comorbidity, the most frequent being hypertension (24.4%) and diabetes (26.8%). The primary clinical symptoms reported included flank pain (24.4%) and urinary urgency (24.8%), while a small percentage (26.8%) of participants were asymptomatic. Additionally, a significant portion (54.4%) of the participants had a family history of renal stones, highlighting the genetic predisposition associated with this condition.

**Table 1:** Percentage of Demographic and Clinical Characteristics

Characteristic	Count	Percentage (%)
Gender (Male)	131	52.4
Gender (Female)	119	47.6
Mean Age	-	35.05
Mean BMI	-	26.93
Smoking Status (Yes)	132	52.8
Alcohol Use (Yes)	129	51.6
Comorbidities (Diabetes)	67	26.8
Comorbidities (Hypertension)	61	24.4
Comorbidities (Both)	60	24.0
Family History (Yes)	136	54.4
Clinical Symptoms (Flank Pain)	61	24.4
Clinical Symptoms (Urinary Urgency)	62	24.8
Clinical Symptoms (None)	67	26.8

Stone characteristics and diagnostic findings

All individuals in the study were diagnosed with renal stones, with sizes ranging from 5.2 mm to 19.9 mm (average = 11.97 mm, SD = 4.15). The stones were found in the kidney (46.8%) and ureter (53.2%). Composition analysis indicated that calcium oxalate stones were the most prevalent (34.8%), followed by uric acid stones (37.6%) and struvite stones (27.6%). Hydronephrosis, a frequent complication associated with urinary obstruction, was noted in 48.4% of the cases, underscoring the severity of obstruction within this group. Additionally, nearly half of the participants (52.4%) had a history of previous renal stone episodes, suggesting a pattern of recurrence in this population. Diagnostic imaging techniques predominantly included ultrasound (51.2%) and CT scans (48.8%), which offered detailed information regarding stone location, size, and related complications. Percentages are shown in Table 2.

**Table 2:** Stone Characteristics and Diagnostic Findings

Characteristic	Count	Percentage (%)
Stone Location (Kidney)	117	46.8
Stone Location (Ureter)	133	53.2
Mean Stone Size	-	11.97
Stone Composition (Calcium Oxalate)	87	34.8
Stone Composition (Uric Acid)	94	37.6
Stone Composition (Struvite)	69	27.6
Hydronephrosis Presence (Yes)	121	48.4
Previous Stone Episodes (Yes)	131	52.4
Diagnostic Method (Ultrasound)	128	51.2
Diagnostic Method (CT Scan)	122	48.8

Treatment interventions and procedure outcomes

The research evaluated two lithotripsy methods: SWL and URSL. Each treatment cohort consisted of 125 participants. The mean duration of the procedures was around 74.73 minutes (SD = 25.73), with times varying from 30 to 119 minutes, indicating differences influenced by the nature of the stones and the technique employed (Table 3).

**Table 3:** Treatment Interventions and Procedure Outcomes

Characteristic	Count	Percentage (%)
Lithotripsy Procedure (Shockwave)	125	50.0
Lithotripsy Procedure (Ureteroscopic)	125	50.0
Mean Procedure Duration	-	74.73
Complications (None)	89	35.6
Complications (Bleeding)	85	34.0
Complications (Infection)	76	30.4
Follow-up Duration (Mean)	-	5.91
Recurrence Status (Yes)	123	49.2

Complications and follow-up

Complications occurred in 64.4% of the cases, with the most frequently reported issues being bleeding at 34.0% and infection at 30.4%. Conversely, 35.6% of patients did not experience any complications, suggesting a level of safety associated with these procedures when conducted in controlled environments. The follow-up period ranged from 1 to 11 months, averaging 5.91 months (SD = 3.21). Notably, stone recurrence was noted in 49.2% of patients, underscoring the necessity for ongoing monitoring after treatment.

Lab results and comorbidities

Laboratory results offered a further understanding of the patients’ health status. The average white blood cell (WBC) count was 7307 cells/µL (SD = 1919.85), while serum creatinine and calcium levels averaged 1.03 mg/dL and 9.47 mg/dL, respectively. These findings suggest that renal function was generally stable among the group (Table 4).

**Table 4:** Lab results and comorbidities NSAIDs: Nonsteroidal anti-inflammatory drugs

Characteristic	Count	Percentage (%)
Mean WBC Count	-	7307.39
Mean Serum Creatinine	-	1.03
Mean Calcium Levels	-	9.48
Medication History (Painkillers)	78	31.2
Medication History (Antibiotics)	90	36.0
Medication History (None)	82	32.8
Current Medications (NSAIDs)	90	36.0
Current Medications (Alpha Blockers)	71	28.4
Current Medications (None)	89	35.6

Treatment outcomes

The main objective of stone removal was successfully met in every instance. Nevertheless, the secondary outcomes, such as complication rates, post-procedural discomfort, and recurrence, exhibited variability. An examination of these secondary outcomes revealed that 34.0% of patients reported post-procedural pain, while 28.0% experienced a recurrence of stones, indicating persistent risks linked to both methods.

Statistical analysis of treatment effectiveness

Chi-square tests were utilized to assess the distribution of categorical variables, including gender, smoking habits, alcohol consumption, family medical history, clinical symptoms, stone location, and treatment outcomes. The results revealed no statistically significant differences in the distribution of these variables among the treatment groups, reinforcing the hypothesis that the groups were demographically and clinically similar at baseline. Importantly, there was no significant difference in treatment success rates between SWL and URS (p > 0.05) (Table 5).

**Table 5:** Statistical Analysis of Treatment Effectiveness

Characteristic	p-Value	t-Value / Chi-Square / F-Value	Significance
Chi-Square Significance (Gender)	0.448	Chi-Square = 0.597	Not Significant
Chi-Square Significance (Smoking Status)	0.376	Chi-Square = 2.067	Not Significant
Chi-Square Significance (Alcohol Use)	0.613	Chi-Square = 0.453	Not Significant
Chi-Square Significance (Complications)	0.587	Chi-Square = 0.317	Not Significant
Chi-Square Significance (Recurrence)	0.800	Chi-Square = 0.061	Not Significant
Mann-Whitney (Age by Gender)	0.499	Mann-Whitney U = 1760.5	Not Significant
Mann-Whitney (BMI by Gender)	0.983	Mann-Whitney U = 1977.0	Not Significant
Mann-Whitney (Stone Size by Gender)	0.686	Mann-Whitney U = 1749.0	Not Significant

The Mann-Whitney U test was performed to evaluate variations in continuous variables such as age, BMI, stone size, procedure duration, and laboratory results between gender categories. The analysis revealed no statistically significant differences based on gender for these variables (p > 0.05), indicating that treatment outcomes are consistent for both male and female patients (Table 5).

Re-treatment requirements

The SWL procedures in this study were conducted using the Dornier Compact Delta II lithotripter, a reliable and widely used model for the non-invasive treatment of renal stones. On average, 2,500 shocks were delivered per session, with adjustments made based on stone size, composition, and patient tolerance. The URSL procedures employed both rigid and flexible ureteroscopes. Rigid ureteroscopes were utilized for stones located in the distal ureter, while flexible ureteroscopes were preferred for proximal ureteral and intrarenal stones, allowing for enhanced maneuverability and access.

Re-treatment was required in 128 (51.2%) participants overall. When analyzed by treatment modality, 63 (50.4%) of the SWL cases and 65 (52%) of the URS cases required additional interventions due to incomplete stone clearance or recurrence. Despite the similar overall rates, the reasons for re-treatment differed, reflecting variations in the mechanisms of each technique. Stone clearance success rates were higher with URS compared to SWL, as the retained stone rates were 27 (21.6%) for SWL and 12 (9.6%) for URS. This disparity underscores the mechanical advantage of URS, which allows for direct stone fragmentation and retrieval, particularly in challenging anatomical locations.

Re-treatment was necessary for 51.2% of participants, indicating that further interventions were needed when the initial treatment failed to achieve complete stone clearance or when recurrence occurred (Table 6). The rates of re-treatment were similar between the SWL and URS groups, demonstrating the comparable efficacy of both approaches.

**Table 6:** Re-treatment Requirements

Characteristic	Count	Percentage (%)
Re-treatment Requirement (Yes)	128	51.2
Re-treatment Requirement (No)	122	48.8

## Discussion

In this prospective cohort study, patients in Pakistan ranging in age from 18 to 55 years old were evaluated for their likelihood of undergoing either SWL or URSL to remove kidney stones. Due to the high rates of stone clearance achieved by both treatments, the study did not find any significant differences in primary outcomes. Complication rates and recurrence, two secondary outcomes, followed comparable trends in the two groups. In 64.4% of cases, complications were noted; the most common of these were infections (30.4%) and bleeding (34.0%). Half of the patients needed to be treated again because 49.2% had recurrence [10-12]. Overall, SWL and URSL had similar safety and efficacy profiles, with some variation in procedure duration and post-procedure discomfort.

Significant clinical implications for the management of renal stones in this population are borne out by the findings. When it came to initial stone clearance, both SWL and URSL worked well, giving patients options for treatment that were personalized according to their preferences and clinical factors [13-15]. Both approaches have the potential to be effective in the long run due to the low rates of complications and recurrence. These findings further emphasize the significance of addressing long-term risks, like recurrence and re-treatment requirements, by means of continuous monitoring and preventative measures. The fact that SWL and URSL do not differ significantly in terms of treatment success rates further supports the idea that they are complementary methods rather than competing ones [16, 17].

Previous studies that compared SWL and URSL found similar results. For stones less than 2 cm in size, prior meta-analyses have shown that the two methods achieve similar stone clearance rates. Nonetheless, various studies have brought attention to variations in the types of complications: A greater likelihood of residual fragments is linked to SWL, whereas ureteral injuries can result from URSL [18, 19]. Findings that show improvements in technology and operator competence have reduced risks for both procedures are consistent with this study's conclusion that there was no statistically significant difference in the complication rates between the groups. It is worth mentioning that the recurrence rate of 49.2% is in line with worldwide trends, which highlights how common renal stones are and the importance of focused prevention efforts [20].

The prospective design of the study is one of its strongest points since it reduces the possibility of recall bias and permits careful tracking of treatment results over time. Accurate evaluation of stone properties and treatment effectiveness was achieved through the use of standardized diagnostic methods, such as CT scans and ultrasounds [21, 22]. The results are more likely to be accurate because the cohort was representative of the population and because the treatment groups had comparable demographic and clinical variables. Having said that, there are a few restrictions. The average duration of follow-up is 5.91 months, which might not be long enough to detect differences in outcomes, especially recurrence rates, over the long term. The study also did not account for other variables, such as patients' compliance with medical and dietary recommendations, even though it did account for factors like age, stone size, and comorbidities. Patients who opted for URSL may have had more severe or symptomatic stones, which could lead to selection bias if treatment groups are dependent on patient choice. In addition, the relatively high re-treatment rate of 51.2% indicates that some patients may not have benefited enough from the first treatments, which calls for additional research into treatment protocols and success criteria [23-25].

To better understand the long-term efficacy and recurrence patterns of SWL and URSL, future research should incorporate longer follow-up periods to address the limitations of this study. To compare the two methods more accurately, randomized controlled trials should be conducted to remove selection bias. Research that divides patients into groups according to stone size, location, and composition would provide more specific information about which patients get the best results from each treatment. Particularly in contexts with limited resources, investigating cost-effectiveness analyses could shed light on how best to distribute healthcare funding. It is also important to study new lithotripsy technologies, like third-generation SWL devices or laser-assisted URSL, to see how they affect safety and effectiveness. The high recurrence rates seen in this study highlight the need for future research to incorporate preventive strategies, such as dietary modifications and pharmacological interventions.

## Conclusions

This prospective cohort study showed that there are no significant variations in primary outcomes, such as stone clearance rates, between URSL and SWL, two effective methods for treating renal stones. Although SWL was less invasive, URSL was just as effective, especially for bigger or more complicated stones. Both groups experienced manageable complications like bleeding and infection, and the rates of recurrence were also comparable. According to these results, both approaches have merit, and the choice of treatment should be based on the specifics of each patient's case. Improving patient selection and maximizing long-term results requires additional research.
